# Suicidal behavior in homeless population and its relationship with experienced aggressions: A seven-year longitudinal study.

**DOI:** 10.1192/j.eurpsy.2024.720

**Published:** 2024-08-27

**Authors:** F. Calvo, R. Alfranca, X. Solench-Arco, C. Giralt, I. Forcada, S. Font-Mayolas

**Affiliations:** ^1^Departament de Pedagogia, Institut de Recerca sobre Qualitat de Vida; ^2^ Institut Català de la Salut, Centre d’Atenció Primària Santa Clara; ^3^ Universitat de Girona; ^4^ Institut Català de la Salut, Centre d’Atenció Primària Blanes 2; ^5^Departament de Psicologia, Institut de Recerca sobre Qualitat de Vida, Girona, Spain

## Abstract

**Introduction:**

Suicide is a serious and complex public health issue that affects millions of people worldwide. Among the most vulnerable populations are homeless individuals (HIs), whose suicide rate is significantly higher than that of the general population.

**Objectives:**

The aim of this study was to analyze mortality and suicidal behavior in a cohort of HIs during a seven-year follow-up. Additionally, the study sought to identify variables linked to mortality in this population.

**Methods:**

The study was conducted in the province of Girona, Spain, and included 154 HIs who were literally experiencing homelessness. Self-report questionnaires were used to gather sociodemographic data, assess suicide risk, and measure the severity of substance dependence. The follow-up was carried out between 2015 and 2022, collecting data on mortality, suicide attempts, episodes of overdose, and violence experienced from public health services (psychiatric and primary health care services).

**Results:**

During the seven-year follow-up, 23 individuals (14.3% of the sample) passed away, with an average age at the time of death of 52.6 years. The main causes of death were cancer, suicide (excluding overdose), and accidental overdose. Methods used for suicide included drug overdose, jumping, and vein slashing. All deceased individuals had scores above the threshold on the Plutchik Suicide Risk Scale and had reported previous suicide attempts.

Individuals who experienced violence during the follow-up period exhibited more severe suicidal ideation, more suicide attempts, and more non-lethal overdose episodes. Substance dependence, particularly cocaine dependence and dual pathology, was significantly associated with higher mortality.

**Conclusions:**

This study reveals a high mortality rate among HIs, especially due to suicide and accidental overdose. The most significant variables related to mortality were suicidal ideation, the number of previous non-lethal overdoses, and substance use disorders, with cocaine dependence being prominent. The results underscore the need for specific prevention and treatment programs to address suicide risk factors and improve the mental health of homeless individuals. The importance of conducting interventions in specialized centers that detect and address suicide risk in this vulnerable population is also emphasized.

**Disclosure of Interest:**

None Declared

